# Editorial: Red Blood Cells at the Mount of Truth: Highlights of the 22nd Meeting of the European Red Cell Research Society

**DOI:** 10.3389/fphys.2020.607456

**Published:** 2020-11-19

**Authors:** Anna Bogdanova, Lars Kaestner

**Affiliations:** ^1^Red Blood Cell Research Group, Institute of Veterinary Physiology, Vetsuisse Faculty and the Zurich Center for Integrative Human Physiology (ZIHP), University of Zurich, Zurich, Switzerland; ^2^Theoretical Medicine and Biosciences, Saarland University, Homburg, Germany; ^3^Experimental Physics, Saarland University, Saarbrücken, Germany

**Keywords:** red blood cells, anemia, technology development, adaptation, modeling

This topic is a result, and in memory of the 22nd meeting of European Red Cell Society (ERCS) in March 2019. The meeting venue, the Stefano Franscini Congress Center Monte Verità (Mount of Truth) at Ascona in Switzerland, is famous amongst creative and artistic people for over a century. The magic of the Monte Verità inspired, and its Bauhaus architecture provided shelter to theosophists, reformers, anarchists, communists, psychoanalysts, writers, poets, artists, dancers, emigrants of both world wars (Bollmann, 2019), and to approximately 100 “ERCS family members” from all over the world Figure 1. Traditionally, the ERCS meetings create a lively and informal platform bringing together the scientists studying red blood cells (RBCs), practicing hematologists, and those that develop new tools to study RBCs and diagnose blood diseases (Bogdanova et al., 2020). Looking at the problems from various perspectives, finding common interdisciplinary language, brain-storming, are the features of ERCS meetings since 1976. Each of the articles of this collection is based on a meeting's presentation.

**Figure 1:**
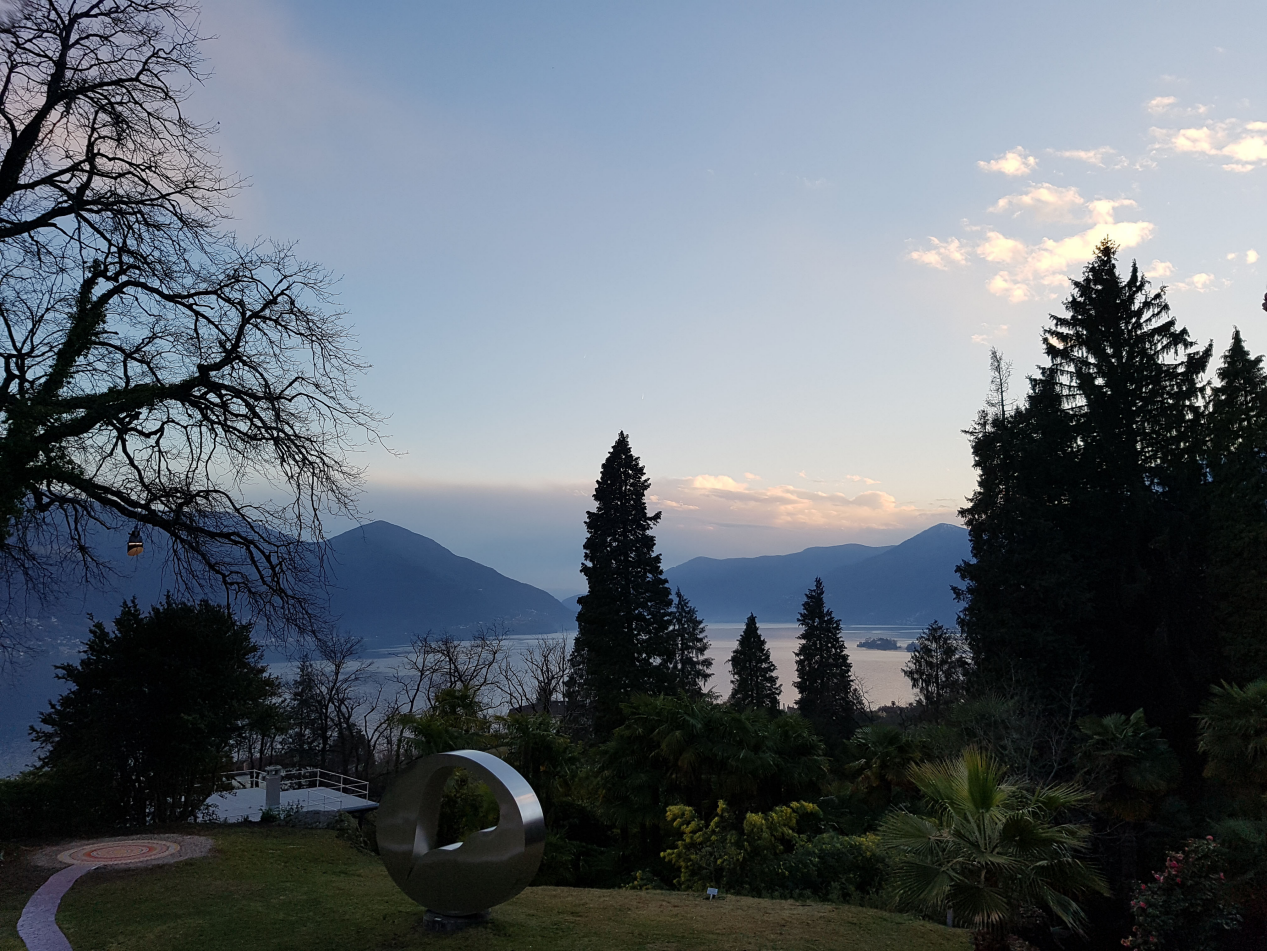
After the sessions: View to the Lake Maggiore from Monte Verita in March 2019.

Adaptation of healthy humans to environmental challenges such as high altitude and endurance sport is mirrored by the changes in RBCs. Such adaptations include broad metabolic remodeling that occurs in elite cyclists (San-Millán et al.). Less extreme, modest physical exercises induces adaptive processes that impact RBC rheology and may be used to improve quality of life of patients with sickle cell disease (SCD) (Nader et al.). Further adaptations, including those to high altitude, result in production of subsets of RBCs that differ from the ones produced on a regular basis (Bogdanova, Kaestner et al.).

A systemic approach was used to analyze the processes in the organism that are associated with stimulation of erythropoiesis by hypoxia or ischemia or by administration of recombinant erythropoietin (Epo) (Suresh et al.). Recent studies revealed a number of tissues apart of erythroid precursor cells in the bone marrow that express Epo receptors and respond to an increase in Epo turning this cytokine to a regulator of adipogenesis, osteogenesis, brain and heart development (Suresh et al.). Further cross-talk was shown between megakaryocytes, and erythroid lineage that rely on glutamatergic signaling via N-methyl D-aspartate receptors to differentiate, proliferate and survive in the bone marrow (Kalev-Zylinska et al.). Better understanding of this receptor-mediated signaling pathway may help in treatment of leukemia and other forms of cancer that require glutamatergic stimulation to support high proliferative activity (Kalev-Zylinska et al.). Recent developments of our understanding of the role of hepcidin in regulation of iron uptake and, hence, erythropoietic activity and progression of anemia is reviewed by Pagani et al. along with therapeutic strategies based on modulation of hepcidin levels in the organism. Summarized by Salinas Cisneros and Thein, recent advances in therapeutic options for the most wide-spread hereditary anemia, SCD are discussed. They range from gene editing and bone marrow transplantation to an increasing number of drugs for symptomatic treatment of the disease. Furthermore, specially designed physical exercises are suggested by Nadir et al. as a complementary treatment that may help to improve condition of SCD patients.

From the physicist's point of view, the RBC can be regarded as a concentrated solution of hemoglobin and other minor components of the cytosol surrounded by plasma membrane (Svetina). A review article of Svetina shows how recently discovered mechano-sensitive Piezo1 ion channels contribute to the feedback between cell volume and its shape. The article gives an overview of development of theoretical models describing RBC shape and volume changes and introduces the problems that still have to be resolved to understand the mechanisms in control of RBC reversible and irreversible shape transitions, that define RBC rheology and functionality, but also describe cell-to-cell differences in shape, density, and deformability. Causes of intercellular heterogeneity for RBCs are discussed in detail in a review article of Bogdanova, Kaestner et al.. Those include the cell age, and the changes in micro- and macro-environment of erythroid precursors as well as of circulating RBCs. Cloos et al. have visualized the cross-talk between the lipid domains and proteins in the course of vesiculation of stored RBCs. The role of ATP depletion, oxidative stress and Ca^2+^ accumulation as triggers of redistribution of lipids within sub-microdomains containing sphingomyelin, ceramide and cholesterol and the loss of cholesterol was explored over 3 weeks of storage.

The needs for RBC analysis, the new concepts addressing these needs and instrumental diagnostic and monitoring solutions to them are reviewed by Kaestner and Bianchi. More examples of novel approaches to explore RBC include lipidomics (Cloos et al.), metabolomics (San-Millán et al.) and the need to look at single cells and RBC sub-populations (Bogdanova, Kaestner et al.), and use theoretical modeling to amplify the predictive power of experimental data (Svetina).

As expected, participants left the meeting inspired and ready to try new approaches, test new ideas and prepare for the next meeting to come in 2020 (virtually) and in 2022 (in person).

## Author Contributions

Both authors contributed to compiling, discussing, and editing the editorial.

## Conflict of Interest

The authors declare that the research was conducted in the absence of any commercial or financial relationships that could be construed as a potential conflict of interest.
